# Stirring inclusion: how diversity-oriented HR practices boost adaptive performance in Greece's food & beverage industry

**DOI:** 10.3389/fpsyg.2026.1768113

**Published:** 2026-02-19

**Authors:** Kleanthis Katsaros, Olga Malisova, Vera Lazanaki, Evdokia Tsoni

**Affiliations:** 1 Department of Food Science & Technology, School of Agricultural Sciences, University of Patras, Agrinio Campus, Patras, Greece; 2 Department of Marketing and Communication, School of Business, Athens University of Economics and Business, Athina, Greece; 3 Department of Management Science and Technology, School of Business, Athens University of Economics and Business, Athina, Greece

**Keywords:** adaptive performance, diversity, inclusion, organizational change, social exchange theory, work engagement

## Abstract

Drawing on social exchange theory and the norm of reciprocity, the paper suggests a moderated mediation model to examine the role of employee perceived inclusion and work engagement in the relationship between diversity-oriented HR practices (DHRP) and employee adaptive performance. This study draws on data from 415 employees in the Greek food and beverage industry and their supervisors in three sequential phases. During the first phase, employees assessed their organizations' DHRP. In the second phase, employees' perceived inclusion and work engagement were examined, and in the third phase, supervisors assessed employees' level of adaptive performance. The results reveal that perceived inclusion mediates the relationship between DHRP and adaptive performance. Further, they provide support that work engagement moderates the relationship between perceived inclusion and employee adaptive performance, as well as the indirect relationship between DHRP and employee adaptive performance through inclusion, such that the positive relationships are stronger once work engagement is higher. The research findings show that by implementing DHRP and procedures, leaders and managers can increase employees' sense of inclusion and work engagement, ultimately boosting their adaptive performance during change.

## 1 Introduction

“*In diversity there is beauty and there is strength…”* Maya Angelou

In today's increasingly diverse workplace, organizations are continually seeking ways to foster inclusion, enhance employee engagement, and drive performance outcomes, mostly in times of organizational changes (Jerónimo et al., 2022; Katsaros, 2024a). The increasing acknowledgment of diversity as a critical strategic asset has led many organizations to employ diversity-oriented human resource practices (DHRP). That is, they can encourage innovation and creativity by integrating diverse perspectives, thereby improving problem-solving and producing unique solutions. At the same time, the diversity, equity, and inclusion (DEI) literature underscores the importance of workplace diversity and investigates how individual, contextual, and situational factors, along with underlying mechanisms, may shape diversity outcomes (Roberson et al., 2017). While the link between such practices and employee performance has been extensively studied (e.g., Ellemers and Rink, 2016; Gomez and Bernet, 2019), the way through which DHRP may influence employee adaptive performance remains quite underexplored. Although DHRP can foster greater adaptability, resilience, and openness to learning among employees, thereby enhancing their overall adaptive performance in the workplace, the precise mechanisms driving this effect remain unclear. This study seeks to address this theoretical gap by examining the role of employee perceived inclusion and work engagement in the relationship between DHRP and employee adaptive performance (EAP).

Grounded in social exchange theory (Blau, 1964) and the norm of reciprocity (Gouldner, 1960), the study proposes a moderated mediation model to better understand how inclusive work environments and engaged employees contribute to adaptive performance. Specifically, it explores how employees' perceptions of inclusion act as a mediator between DHRP and EAP, while work engagement is hypothesized to moderate the strength of these relationships. That is, on the one hand, it is suggested that diversity and inclusion are mutually dependent, with diversity acting as an fundamental precursor to inclusion (Nguyen et al., 2024) and on the other hand, inclusion initiatives and practices can help employees remain resilient and maintain focus during periods of change (Katsaros, 2025). Furthermore, HR researchers widely agree that engaged employees not only feel a strong sense of inclusion and belonging at work (Jerónimo et al., 2022) but also play a decisive role during turbulent times by providing the necessary personal resilience, and persistence (Chanana and Sangeeta, 2021). The current study offers valuable insights into the dynamics that can enhance employee performance in response to diversity initiatives based on data collected from 415 employees in the Greek food and beverage industry. The findings note the key role of perceived inclusion and work engagement in strengthening the positive outcomes of DHRP, and they offer both theoretical insights and practical guidance for managers and leaders aiming to enhance employee performance during organizational change.

This study enriches the existing body of literature by offering three primary contributions. First, it addresses the call for further empirical evidence on employee adaptive performance (Park and Park, 2021; Vakola et al., 2021). To this point, there are more than a few studies that examine employee adaptive performance (Park and Park, 2019), but there are rather few that examine the relationship between diversity and employees' ability to adjust to changes in their workplace (e.g., Devassy and Jindal, 2024). This is quite confusing, as organizations may have the opportunity to unlock and elevate the full potential of their people and stay ahead in a endlessly changing world by adopting diversity, equity, and inclusion (Weber and Gaggiotti, 2024). This contribution holds value for both HRM researchers and practitioners. Second, while researchers have sufficiently explored how change recipients respond to change initiatives (e.g., Oreg et al., 2023), unexpectedly, they haven't fully expanded on how diversity-oriented approaches may endorse participative, active and helpful behaviors in times of change (e.g., Liu et al., 2023). Failing to do so may result in ineffective change management efforts, as employees who feel included and engaged are more likely to embrace change rather than resist it (Khaw et al., 2023). Third, this research contributes to the HRM and change management literature by highlighting the importance of employees ‘perceived inclusion and work engagement in changing and diverse organizational settings. The research underscores these factors as essential for driving positive outcomes in organizational transformation, making them key elements to consider in change management strategies.

## 2 Literature review and hypotheses development

### 2.1 Diversity-oriented HR practices and employee adaptive performance

The HRM literature provides robust evidence that HR practices must account for workforce diversity and create opportunities derived from their differences (e.g., Guillaume et al., 2017; Wang et al., 2020). That is, leveraging diversity helps organizations better respond to market changes, attract top talent, and improve decision-making, ultimately contributing to their competitiveness and sustainability. Accordingly, diversity-oriented HR practices (DHRP) refer to practices that directly try to communicate to the employees the organization's values regarding diversity and inclusion (Jehn and Bezrukova, 2004), and thus, they may strengthen the value of workforce diversity for an organization (Ashikali and Groeneveld, 2015). These practices aim to create an inclusive environment that values and respects individual differences, leading to a more engaged and innovative workforce. As a result, these human-oriented practices may positively influence employees' job satisfaction, creativity, work engagement, and organizational commitment, as well as moderate their turnover intentions (Luu et al., 2019; Madera et al., 2017; Trong Tuan, 2020). They aim not only to foster diversity and inclusion, but also to transform these differences into drivers of organizational success (e.g., financial performance, corporate social responsibility, innovation, satisfaction and loyalty of diverse customers, competitive edge; Gomez and Bernet, 2019; Manoharan and Singal, 2017). DHRP are characterized by empathy, anthropocentric orientation, respect, justice, social-emotional support, and trust (Meena and Vanka, 2017), and hence, they may assist employees navigate the frictions that come with organizational change (Gomez and Bernet, 2019). Quite similarly, Ford et al. (2008) suggest that a change agent who may repair damaged relationships and restore trust both before and during a change (i.e., by embracing employees' diversity and inclusion) is less probable to encounter resistance to change than an agent who doesn't employ such anthropocentric approaches during change efforts.

Change management literature indicates that employee adaptive performance is essential for every organizational change endeavor (e.g., Vakola et al., 2021). That is, when employees are adaptable, they are more likely to embrace change, navigate uncertainty, and contribute to the smooth implementation of new strategies or processes. Adaptive performance refers to employees' adaptability to the changes and evolutions in their work environment (Park and Park, 2019). Jundt et al. (2015) describe it as an individual behavior that can be displayed both in anticipation of and in reply to a change effort. On the individual level, this may foster several beneficial outcomes, including heightened work engagement (Kaya and Karatepe, 2020) and greater job satisfaction (Marques-Quinteiro et al., 2019). It may also cause significant organizational outcomes such as managing change and organizational learning (Dorsey et al., 2017). Adaptive performance studies have mainly focused on skill-based adaptation (Jundt and Shoss, 2023); however, adaptive performance can also encompass adjustments in interpersonal and emotional behaviors aligned with organizational change. This study examines adaptive performance as the cognitive, emotional, and behavioral efforts individuals employ to navigate change.

Provided that we are living in an era of unprecedented challenges and opportunities, only a handful of studies have investigated how diversity and inclusion affect employee responses to change (e.g., Moncloa et al., 2019). That is, diversity and inclusiveness within organizational settings may act as powerful tools in managing VUCA (Volatility, Uncertainty, Complexity, and Ambiguity) by enhancing adaptability, innovation, and resilience in the face of rapid and unpredictable changes (O'Donovan, 2018). That is, a diverse and inclusive workplace may promote novelty and creativity and bring about new perspectives and approaches to challenges and/or opportunities (Abbas, 2023). Within this context, Rodriguez (2022) notes that positive transformations need the infusion of new perspectives, and thus, initiatives that boost diversity, equity, and inclusion may cultivate organizational agility and serve as transformative forces in driving change, and Li et al. (2020) have found that that effective workforce diversity management is positively associated with employees' job match, job satisfaction, and job performance.

Further, the social exchange theory suggests that when employees experience positive behavior from others, they are likely to reciprocate with actions of comparable significance (Blau, 1964), and the norm of reciprocity posits that when employees are treated positively by their organization, they experience an inherent obligation to reciprocate through constructive workplace attitudes (Gouldner, 1960). Thus, if employees receive respect, honesty, fairness, empathy, justice, social-emotional support, and trust from their organizations during change (Meena and Vanka, 2017), they will feel compelled to reciprocate by exhibiting positive behaviors that support organizational change (Garba et al., 2018). Overall, DHRP are expected to be positively related to employee adaptive performance because they may create a workplace where employees are more adaptable, innovative, and resilient (Showkat and Misra, 2022), which are all key components of strong adaptive performance. Drawing on the above rationale, we hypothesize:

*H1. Diversity-oriented HR practices are positively related to employee adaptive performance*.

### 2.2 The mediating role of employee perceived inclusion

The diversity, equity, and inclusion (DEI) literature highlights that inclusion at the individual level reflects employees' perceptions of organizational fairness and the recognition of both their belongingness and uniqueness (e.g., Downey et al., 2015; Mor Barak et al., 2022). It refers to the degree to which employees feel valued, appreciated, and accepted within their organization and it encompasses employees' sense of belongingness, fairness, and being part of a supportive, inclusive workplace culture. That is, when employees perceive high levels of inclusion, they sense that their efforts are appreciated and that they are empowered to take part wholeheartedly in organizational initiatives. Hence, it reflects whether employees feel appreciated and supported in their work environment and whether they believe they can freely contribute to the organizational goals. In this context, Shore et al. (2011), by using Brewer's (1991) optimal distinctiveness theory, conceptualized employee perception of inclusion, on the one hand, as satisfying the need for belongingness (e.g., recognition, acceptance by others, and a sense of shared identity), and on the other hand, a counteracting need for uniqueness (e.g., the desire to sustain a distinct personal identity). Employees in organizations perceived as inclusive, with supportive HR practices, tend to demonstrate greater psychological safety (i.e., lower psychological distress; Aslan et al., 2021), higher job satisfaction (Brimhall and Mor Barak, 2018), stronger organizational citizenship behaviors (Aboramadan et al., 2021), enhanced work engagement (Katsaros, 2024a), and improved job performance in contexts of change (Vakira et al., 2023). Further, workplace inclusion is also related to workplace happiness (Mousa, 2020) and culturally agile organizations (Selzer and Foley, 2018). Thus, it is not surprising that more than 90% of Fortune 500 companies have instituted formal DEI initiatives aligned with critical organizational goals, and that over 70% of Fortune 1,000 companies maintain formal DEI programs (Mondal, 2021). Numerous studies today try to identify which HR practices may enhance employees' perception of inclusion. Chaudhry et al. (2021) argue that inclusion practices encompass fairness, belongingness, uniqueness, and a diverse workplace climate, while Nguyen et al. (2024) describe an inclusive climate as one characterized by fair practices, the integration of differences, and participation in decision-making. Accordingly, quite a few inclusive practices have been proposed to build such a climate, including DEI training (Leslie, 2019), the establishment of employee resource groups or identity-conscious programs (Li et al., 2019), and the implementation of reverse mentoring (Chaudhuri et al., 2022).

Given that we are in an era of constant change, it is becoming increasingly essential to identify the factors that drive successful adaptation. Many organizations today try to employ inclusive approaches in the workplace that may improve employees' change related attitudes and behaviors (Katsaros, 2024a). That is, prior research suggests that inclusive HR practices can enhance employee wellbeing and foster innovative behavior (Choi et al., 2017), while also stimulating engagement during organizational change (Zeng et al., 2020). For example, drawing on affective events theory, Qurrahtulain et al. (2022) found that vigor at work mediates the relationship between inclusive leadership and adaptive performance. Similarly, Bataineh et al. (2022), in a study of 169 nurses, demonstrated that inclusive leadership exerts a direct and significant predictive effect on adaptive performance, as well as an indirect effect through innovative work behavior. To sum up, on the one hand, it is suggested that diversity and inclusion are interdependent, with diversity serving as a vital precursor to inclusion (e.g., without diversity in the first place, there is nothing to include) and inclusion being a vital antecedent for realizing the benefits of diversity (e.g., individuals are given the opportunity to contribute their unique perceptions, opinions, skills, and talents; Nguyen et al., 2024; Oswick and Noon, 2014). On the other hand, it is suggested that should leaders and managers employ inclusive strategies, practices, and procedures, they are expected to enhance employees' adaptive performance in the context of organizational change (Bataineh et al., 2022). Thus, diversity-oriented HR practices drive employee perceived inclusion, which enhances employee engagement, trust, and psychological safety, ultimately fostering adaptive performance in changing environments. Based on the above rational and research findings, we suggest that employee perceptions of inclusion may serve as a key mechanism for explaining the connection between diversity-oriented HR practices and employee adaptive performance. Therefore, we hypothesize:

*H2. The positive relationship between diversity-oriented HR practices and employee adaptive performance is mediated by employee perceived inclusion*.

### 2.3 The moderating role of work engagement

Employees' work engagement is considered as a critical factor in contexts of organizational change, as it supplies the personal energy, focus, resilience, and persistence needed to navigate transitions (Vakola et al., 2021). It is a positive, fulfilling, work-related state explained by vigor, dedication, and absorption (Schaufeli et al., 2002). Vigor is a personal resource that embodies the personal drive to channel energy, resilience, and enthusiasm into one's work commitments (Obuobisa-Darko, 2020). It is viewed as a key workplace resource, enabling greater energy, resilience in difficulties, and a sense of job enthusiasm (Timms et al., 2015). Dedication refers to a deep psychological commitment to one's work, characterized by a sense of interest, significance, passion, inspiration, pride, and challenge (Green Jr et al., 2017). Extant organizational research has repeatedly demonstrated its capacity to diminish negative consequences while amplifying positive results (Prodanova and Kocarev, 2022). Further, absorption describes a state of deep focus, concentration, and attachment to one's work (Obuobisa-Darko, 2020). Wood et al. (2020) liken this experience to being so immersed in a task that everything else fades into the background. Empirical evidence further shows that engaged employees tend to display positive work-related attitudes and behaviors (e.g., Brokmeier et al., 2022). Common outcomes include greater job satisfaction, stronger organizational commitment, improved performance, better work–life balance, as well as heightened happiness and enthusiasm (Neuber et al., 2022; Pleasant, 2017; Wood et al., 2020).

There is a high degree of consensus amongst HR researchers that engaged employees usually experience feelings of inclusion and belongingness in the workplace. That is, employees who are actively involved and emotionally invested in their work are more likely to experience feelings of respect and acceptance (Katsaros, 2024a). Within this context, Downey et al. (2015), using a sample of 4.597 health sector employees, have demonstrated that diversity practices foster a climate of trust, which subsequently enhances employee engagement, as well as that inclusion moderates the link between diversity practices and the trust climate. In the same vein, Jerónimo et al. (2022), drawing on a sample of 238 responses, demonstrated a positive correlation between employees' perception of diversity practices and engagement, mediated by perceptions of inclusion. Their findings also indicate that inclusive leadership enhances employees' perception of inclusion. Thus, truly diverse and inclusive workplaces may augment employee engagement (Shuck and Reio Jr, 2014).

Accordingly, work engagement is looked upon as an imperative factor in changing times as it may offer the required energy, concentration, and persistence for effective performance (Katsaros, 2024a). According to van den Heuvel et al. (2020), work engagement plays a crucial role during the initial stages of change, as early and rising levels of engagement forecast employees' attitudes toward change both at the end of the transition and in the longer term. Further, Vakola et al. (2021) uncovered that work engagement may enhance the likelihood that ambivalent employees successfully adapt to organizational change, and Katsaros (2024b), by examining 364 exclusively remote employees, concluded that work engagement serves as a bridge between change self-efficacy, support from supervisors, organizational support, and employees' performance outcomes (i.e., task and adaptive). Drawing on social exchange theory and the norm of reciprocity (Blau, 1964; Gouldner, 1960), which emphasize the exchanges between employees and their organization, diversity and inclusion practices may convey that the organization values employees' mental health, happiness, and wellbeing. Overall, highly engaged employees are more committed to their tasks, and in an inclusive environment, this commitment may boost their ability to adapt to change and overcome challenges. Thus, we hypothesize:

*H3. Work engagement moderates the relationship between employee perceived inclusion and employee adaptive performance, such that the positive relationship is stronger under high work engagement*.

**Figure 1:**
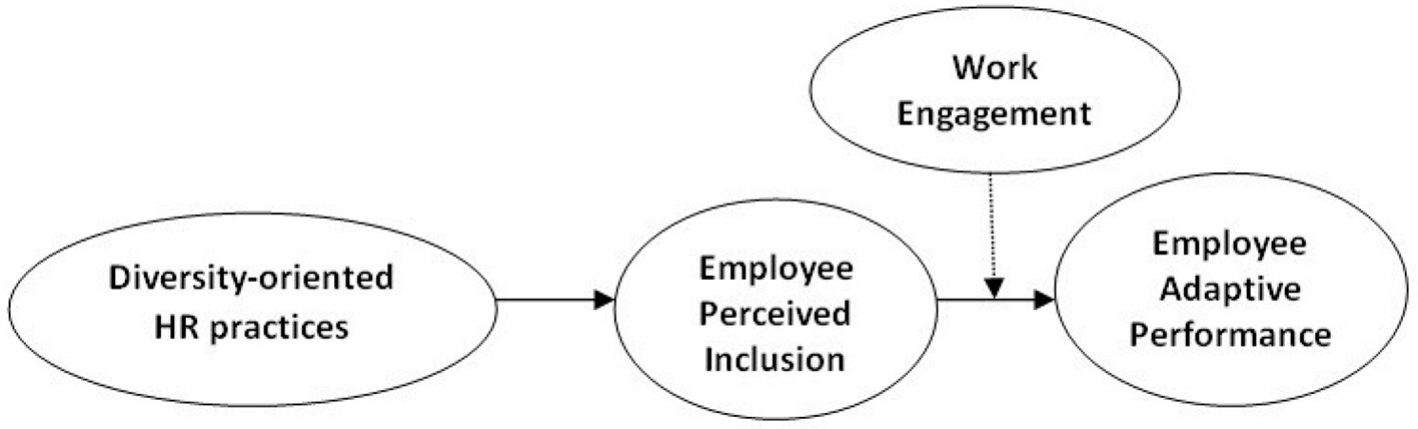
Research model. Source: Authors' work.

The indirect relationship between diversity-oriented HR practices and employee adaptive performance may be influenced by work engagement because engaged employees are more likely to take full advantage of inclusive practices, which then boosts their ability to adapt to changes (Bakker and Albrecht, 2018). When employees are highly engaged, they demonstrate stronger motivation and organizational commitment, which enhances their responsiveness to diversity-oriented HR practices and increases the likelihood of positive performance during periods of change. Thus, we hypothesize:

*H4. The indirect relationship between diversity-oriented HR practices and employee adaptive performance is moderated by work engagement, such that the relationship is stronger under high work engagement than under low one (Figure 1)*.

## 3 Research background

The food and beverage industry has a significant impact on both employment and the Greek economy. It accounts for 28.1% of all enterprises in domestic manufacturing and ranks first among manufacturing sectors in Greece. Specifically, the value of production in the Greek food and beverage industry accounts for 24.4% of the total manufacturing sector, compared to 14.4% in the EU27. Gross value added in Greece amounts to 26.7%, while in the EU27 it stands at 11.5%. The share of turnover in Greece reaches 24.8% of the total manufacturing sector with a turnover of 16 billion euros, compared to 14.0% in the EU27. Also, it is the largest employer in manufacturing, as it employs 37.3% of all employees in the manufacturing sector, compared to 15.7% in the EU27 (data comes from the Eurostat Structural Business Statistics database and refers to the most recent available data, 2021).

Nowadays, the food and beverages industry in Greece faces significant changes as a result of the rising demand for food (e.g., growing population and increasing incomes will lead to higher food demand and as a result meeting this demand sustainably is a major challenge), food affordability (e.g., in 2021, billions of people couldn't afford healthy diets, especially women and rural populations), rising food prices (e.g., the cost of healthy diets increased significantly between 2019 and 2021), land degradation (e.g., soil, grassland, and forest degradation, along with water scarcity and deforestation, threaten environmental and ecosystem services), and food production's environmental impact (e.g., food production contributes significantly to greenhouse gas emissions, primarily from agriculture; Food Drink Europe, 2023).

## 4 Materials and methods

### 4.1 Procedure and participants

The research sample comprised 415 employees and their supervisors in the Greek food and beverage sector, with data obtained in three stages. We used a procedural design suggested by Podsakoff et al. (2003). The study employed a multi-source research design to enhance the robustness and validity of the findings. Participants were assured of confidentiality and academic use only. Data were gathered in three waves, 3 weeks apart, to limit common method bias. During the first phase, employees assessed their organizations' diversity-oriented HR practices. In the second phase, employees' perceived inclusion and work engagement (self-rated) were examined. In the final phase, supervisors evaluated employees' adaptive performance. Utilizing diverse data sources, such as supervisors and employees, contributes to minimizing the potential influence of common method variance (Podsakoff et al., 2003). To enable the alignment of supervisor and employee responses for further analysis, questionnaires included employee identification codes. Participation was voluntary, and all respondents were fully informed of the study's goals via an electronic package outlining directions and instructions. This e-package contained a cover letter and detailed guidance for completion. The research team was present in all phases, online and onsite, to support participants and maintain study quality.

To avoid the common method variance, the confirmatory factor analysis marker technique was used (Williams et al., 2010). Altogether, 456 employees from 29 companies took part in the research study (response rate 64.50%). Subsequently, we removed surveys with missing items, resulting in 415 valid responses for analysis. Further, 55 supervisors took part in the research (response rate 61.11%). The majority of employees were men (67.47%); they had at least a bachelor's degree (54.22%), and they had total experience of 5–10 years (59.52%). Additionally, the majority of supervisors were male (60.00%), they had at least a master's degree (52.73%), and they had total experience of 5–10 years (90.91%). Finally, most of the companies were medium-sized, employing between 50 and 249 people (69%), and 83% of them had been in operation for over 16 years. Additionally, 62% of these companies are considered mature in their lifecycle. Furthermore, all these companies have established HR departments that actively support DEI (Diversity, Equity, and Inclusion) approaches and procedures.

### 4.2 Measures

All questionnaire items were assessed using a five-point Likert-type scale, with response options ranging from “Strongly disagree” (1) to “Strongly agree” (5). Diversity-oriented HR practices (employee-rated) were measured with the 12-item scale developed by Shen et al. (2010). An example item is: “All training programs are open to all employees regardless of personal characteristics and backgrounds”.

Inclusion (employee-rated) was measured with the 18-item scale proposed by Jaiswal and Dyaram (2020; i.e., 11 items from Nishii's (2013) climate for inclusion scale and 7 items from Sabharwal's (2014) organizational inclusion scale pertaining to leadership commitment; 2020). That is, inclusion is a multidimensional concept that can be understood from different perspectives (Felder, 2018). An example item is: “In my organization, leadership creates an awareness and appreciation of individual and cultural differences among employees”.

Work engagement (employee-rated) was measured with the 18-item scale developed by Rich et al. (2010). An example item is: “I am enthusiastic in my job”.

Adaptive performance (supervisor-rated) was measured with the 3-item scale developed by Griffin et al. (2007). An example item is: “This employee has adapted well to the changes in his/her core tasks”.

We first utilized SPSS Statistics v.22.0 to run exploratory factor analyses and compute descriptive statistics. Next, confirmatory factor analysis was carried out with AMOS v.24, followed by hierarchical regression analyses to examine the hypotheses.

## 5 Results

An initial assessment of reliability and validity confirmed that the scales were robust. Cronbach's alpha coefficients for all study variables exceeded the 0.70 benchmark, underscoring strong internal consistency. The factor loadings ranged from 0.80 to 0.90 for the diversity-oriented HR practices' items, from 0.72 to 0.84 for the employee perceived inclusion items, from 0.74 to 0.86 for the work engagement items, and from 0.76 to 0.88 for the adaptive performance items (Table 1).

**Table 1:** Means, standard deviations, and correlations.

**Variables**		**Mean**	**SD**	**Alpha**	**1**	**2**	**3**	**4**	**5**
1.	E.Gender©	1.04	0.52						
2.	E.Work.Exp©	1.22	0.62		0.04				
3.	Diversity-oriented HR practices (*E*)	3.45	0.24	0.77	0.01	0.03			
4.	Employee perceived inclusion (*E*)	4.01	0.35	0.82	0.02	0.05	0.48^**^		
5.	Work engagement (*E*)	3.45	0.41	0.74	0.03	0.12	0.27^*^	0.23^*^	
6.	Adaptive performance (*S*)	4.12	0.44	0.85	0.11	0.07	0.23^*^	0.13	0.54^*^

^*^p < 0.05.

^**^p < 0.01.

*N* = 415.

Source: Authors' work.

©*Categorical variables, (E) Rated by Employees, (S) Rated by Supervisors*.

Results of the regression analysis confirm that diversity-oriented HR practices are positively linked to adaptive performance among employees (*b* = 1.12, *p* < 0.05) and therefore, it provides support for H1 (Table 2).

**Table 2:** Results of regression analysis.

**Variables**	**Adaptive performance**
**Control variables**	
E.Gender	0.44
E.Work.Exp	0.36
**Main variables**	
Diversity-oriented HR practices	1.12^*^
***R*** ^**2**^	0.28
***F***	3.56^*^

^*^p < 0.05.

*N* = 415.

Source: Authors' work.

To evaluate the research model, we used the Structural Equation Modeling (SEM) technique with maximum-likelihood estimation, utilizing the Analysis of Moment Structures software (AMOS, version 24). The analysis was conducted in two phases: first, we constructed and assessed the measurement model through confirmatory factor analysis (CFA), and consequently, SEM was applied to examine the path coefficients. In addition, we employed several well-established model fit indices, including Normed Chi-Square (χ^2^/*df*), Standardized Root Mean Square Residual (SRMR), Goodness of Fit Index (GFI), Comparative Fit Index (CFI), and Root Mean Square Error of Approximation (RMSEA). The model fit indices, presented in Table 3 together with their acceptable thresholds, indicate that the proposed model achieves a satisfactory overall fit.

**Table 3:** Model fit indexes (Source: Authors' work).

**Model fit**	**Mediated model**	**Cutoff Point**	**Reference**
Normed Chi-square (χ^2^/*df*)	1.80	< 3	Qing et al., 2019
Standardized root mean square residual (SRMR)	0.02	< 0.05	Iacobucci, 2010
Goodness fit index (GFI)	0.98	>0.95	Hair, 2011
Comparative fit index (CFI)	0.97	>0.95	Hair, 2011
Root mean square error of approximation (RMSEA)	0.04	< 0.06	Iacobucci, 2010

**Figure 2:**
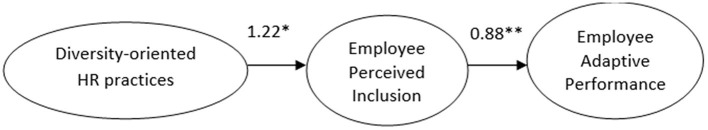
Structural equation modeling results. ^*^p < 0.05, ^**^p < 0.01. Source: Authors' work.

We next conducted a power analysis using the procedure of MacCallum et al. (1996), which yielded power levels above 0.95. This indicates that the sample size was sufficient to minimize the likelihood of Type II errors. Additionally, to further evaluate the validity of the proposed model, we specifically assessed the path coefficients to determine whether the hypothesized relationships are supported by the empirical data. As depicted in Figure 2, employee perceived inclusion mediates the positive relationship between diversity-oriented HR practices and employee adaptive performance (*b* = 0.88, *p* < 0.01). These results are consistent with H2, which was therefore confirmed.

Next, we used regression analysis to examine the moderating role of work engagement on the relationship between employee perceived inclusion and adaptive performance (H3). According to Aiken and West (1991), both employee perceived inclusion and work engagement were centered before running the analyses. As Table 3 exhibits, the interaction term was significant (*b* = 0.88, *p* < 0.05) and explained an additional 16% of the variance in adaptive performance. Next, we used Aiken and West's (1991) procedures to plot the pattern of the significant interaction effects. As Figure 2 shows, the simple slope test further suggests that at high level of work engagement, employee perceived inclusion is positively and significantly related to adaptive performance. However, at low level of work engagement, the relationship between employee perceived inclusion and adaptive performance isn't significant. Therefore, H3 is supported (Table 4).

**Table 4:** Results of hierarchical regression analysis for moderation by work engagement.

**Variables**	**Adaptive performance**		
	**Step 1**	**Step 2**	**Step 3**
**Control variables**			
E.Gender	0.08	0.09	0.10
E.Work.Exp	0.14	0.04	0.14
**Main variables**			
Employee perceived inclusion		0.45^*^	0.34^**^
Work engagement		0.78^*^	0.62^*^
**Interaction term**			
EPI × WE			0.88^*^
***R* ^2^**	0.08	0.22	0.38
***F***	1.74	3.55^**^	4.22^**^
**Δ*R*^2^**	0.08	0.14^**^	0.16^*^

^*^p < 0.05.

^**^p < 0.01.

*N* = 415.

**Figure 3:**
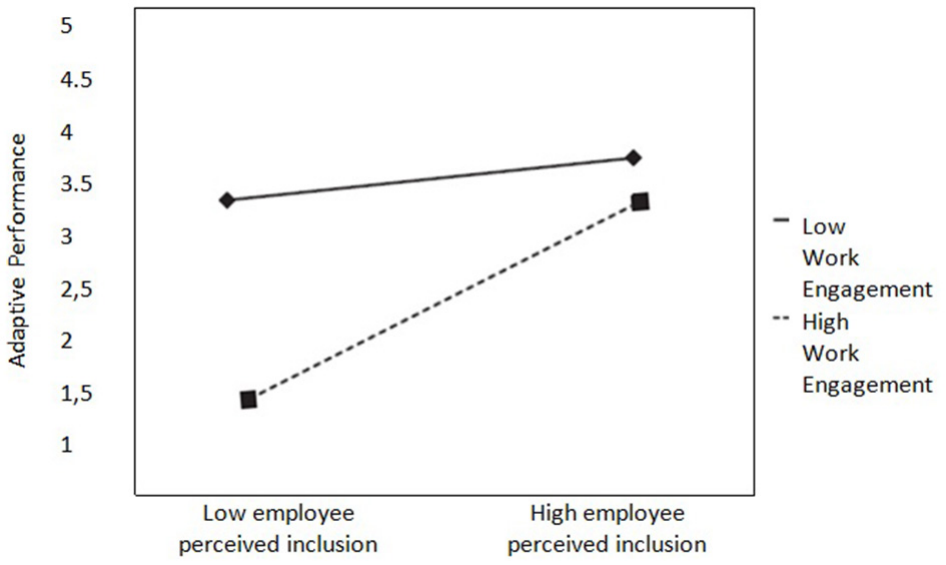
The moderating effect of work engagement on the relationship between employee perceived inclusion and adaptive performance. Source: Authors' work.

Subsequently, the moderated mediation effect predicted in H4 was examined by estimating the conditional indirect effect of diversity-oriented HR practices on adaptive performance via employee perceived inclusion with 95% bootstrapped confidence intervals and 10,000 bootstrap resamples. The 95% bias corrected confidence interval (0.25–2.5) indicated that the conditional indirect effect of diversity-oriented HR practices on adaptive performance via employee perceived inclusion was significant under high work engagement (indirect effect = 1.20) but not significant under low work engagement (indirect effect = 0.30; 95% CI is = −0.05 to 0.95). Thus, H4 is supported (Figure 3).

## 6 Discussion

The research findings support all four hypotheses of the paper. In more detail, they indicate that there is a positive relationship between diversity-oriented HR practices (DHRP) and employee adaptive performance (EAP). Likewise, Qurrahtulain et al. (2022), drawing on affective events theory and social exchange theory, demonstrated that vigor at work mediates the relationship between inclusive leadership and adaptive performance in Pakistan's textile industry. In a complementary study, Katsaros (2024a), based on data from 305 Generation Z employees in the telecommunication sector, concluded that when leaders enhance work engagement and job satisfaction through inclusive practices, they can effectively strengthen employees' adaptive performance.

The research findings also indicate that employee perceived inclusion mediates the relationship between DHRP and EAP. This is not unanticipated considering that, first, employees value workplace happiness, inclusion, and wellbeing (e.g., Adams et al., 2020), and second, HR researchers concur that employee feelings of inclusion may enable his/her participation during change efforts (e.g., Katsaros, 2022). Most important, the analysis revealed that employee work engagement plays a moderating role as it enhances the positive relationship between perceived inclusion and adaptive performance and it also strengthens the indirect effect of diversity-oriented HR practices on adaptive performance through inclusion. Likewise, the DEI literature suggests that, on the one hand, organizations that embrace diversity, equity, and inclusion may positively influence employees' work engagement (e.g., Garg and Sangwan, 2021) and, on the other hand, work engagement may boost the resilience and persistence needed for coping with change effectively (e.g., Katsaros, 2024a; Rafferty and Jimmieson, 2017).

### 6.1 Theoretical implications

From a theoretical perspective, our findings highlight the importance of employee perceived inclusion during change, an aspect that may have been earlier oversaw. The current study suggests that that perceived inclusion serves as a valuable personal resource, strengthened by DHRP and subsequently contributing to higher adaptive performance. This finding also contributes to change management literature's search for mechanisms to achieve greater employee adaptation in changing times (Vakola et al., 2021).

Moreover, the research findings have several implications for sociological and psychological theories such as social exchange theory and the norm of reciprocity. Research emphasizes that perceived inclusion and work engagement critically influence employee behavior, particularly under conditions of ambiguity, uncertainty and organizational transition. Such factors align with social exchange theory, which suggests that perceptions of fairness and inclusion influence employees' commitment and performance. Likewise, reciprocity norms imply that employees engage more positively when they feel valued and included. The findings extend these theories by showing that during times of change, inclusion and engagement become even more crucial in motivating employees, helping organizations navigate through challenging situations.

### 6.2 Practical implications

From an applied standpoint, this study has three important implications for leaders and change management practitioners. First, it notes that employee-perceived inclusion functions as a mediating mechanism between diversity-oriented HR practices (DHRP) and employee adaptive performance (EAP). Consequently, leaders should actively cultivate inclusive and collaborative practices throughout change initiatives. Such practices may include stakeholder engagement, transparent communication, fostering psychological safety, encouraging involvement within work groups, ensuring that employees feel respected and valued, enabling influence in decision-making, demonstrating authenticity, recognizing and advancing diversity, and establishing feedback loops, evaluation, and reflection (Shore et al., 2018). By embedding these practices, organizations can create a supportive climate in which employees remain engaged and valued, and thus, strengthen their adaptive performance during periods of change.

Second, firms and managers should try to increase the level of their employees' work engagement. For example, they should try to promote work-life balance (e.g., flexible work hours, remote work options, vacation time etc.), foster a supportive culture (e.g., inclusion, diversity, equity, anthropocentric and open communication etc.), implement wellness programs (e.g., fitness classes, mental health resources, stress management workshops, counseling services, employee assistance programs etc.), encourage professional and personal development (e.g., training, workshops, career advancement opportunities, mentorship programs etc.), increase job satisfaction (e.g., clear roles and responsibilities, opportunities for autonomy and creativity etc.), cultivate positive relationships (e.g., team-building activities, social events, culture of collaboration etc.), regularly evaluate employees' wellbeing (e.g., surveys, check-ins, feedback etc.) and/or provide healthy work environments (e.g., comfortable and safe physical workspace, ergonomics, lighting, access to natural elements etc) especially in turbulent times (American Psychiatric Association, 2024; Kraszewski, 2024).

Third, organizations may significantly benefit if they train their managers to be supporters and enablers of inclusion in the workplace. According to Roepe (2024) this could happen if they encourage their managers to effectively model the following four behaviors: (a) curiosity (e.g., if an employee is frequently late, instead of assuming that the employee is just indifferent, the manager should ask what is making him/her late and how the manager may help), (b) adaptability (e.g., if a manager says something that doesn't fit well with another employee, the manager should make an apology), (c) personalized approach (e.g., employees need their managers to be empathetic, but that empathy will be diverse for everyone on the team), and (d) employees' ambitions understanding (e.g., managers should ask what their employees aspire to and subsequently, coach them toward that goal/achievement).

### 6.3 Limitations and future research

There are always limitations in scientific research, and this study is no different. The social desirability effect might have had an impact on the participants (Podsakoff et al., 2003). That is, the tendency to respond in a way that will be viewed positively by others, hence not providing completely clear-cut answers. Furthermore, the current study does not adequately reflect the potential complexity of employee adaptive performance, which is unquestionably a multifaceted construct. Accordingly, future research that extends these findings by employing larger and more representative samples would be of unquestionable importance. Another key limitation is that the descriptive correlational design cannot establish causality. Although the study identifies significant associations between diversity-oriented HR practices, inclusion, and adaptive performance, it cannot determine whether these practices actually cause changes in employee outcomes.

Similarly, additional study is required to clarify the connection between diversity-oriented HR practices and employee adaptive performance. For instance, researchers may examine the role of other essential contextual (e.g., opportunities to participate in the change, role clarification, change information, team support, organizational support, change leadership support etc.) and/or personal (e.g., self-motivation, resilience, persistence, self-efficacy, optimism etc.) change resources. The latter would be rather helpful for both the theory and practice of change management. Ultimately, we encourage scholars to draw upon diverse theoretical frameworks to capture the full spectrum of interactions shaping employees' perceptions of workplace inclusion, thereby advancing this field of inquiry.

## 7 Epilogue

The present study shows that diversity-oriented HR practices may enhance employee adaptive performance by fostering a stronger sense of inclusion. Concurrently, it demonstrates that work engagement may positively influence these positive effects. Further, perceived inclusion has emerged as a key psychological mechanism that explains how inclusive practices translate into adaptive behavior during changing times. Overall, the findings clarify why and when diversity-oriented HR practices matter for employee adaptation, contributing to diversity, inclusion, and change management research. For practitioners, the results underscore the value of cultivating inclusive climates and supporting employee engagement to strengthen organizational resilience in times of change.

## Data Availability

The datasets presented in this article are not readily available because datasets generated and/or analyzed during the current study are not publicly available due to privacy and ethical restrictions. In accordance with the informed consent process and agreements made with participants and their organizations, data sharing was not permitted. Requests for further information about the study may be directed to the corresponding author. Requests to access the datasets should be directed to klekatsaros@upatras.gr.
